# An occasional diagnosis of myasthenia gravis - a focus on thymus during cardiac surgery: a case report

**DOI:** 10.1186/1749-8090-4-55

**Published:** 2009-10-07

**Authors:** Marco Agrifoglio, Fabio Barili, Luca Dainese, Antioco Cappai, Faisal H Cheema, Paolo Biglioli

**Affiliations:** 1 Department of Cardiovascular Surgery, University of Milan, Via Parea 4, 20138 Milan, Italy; 2 Department of Cardiovascular Surgery, S Croce Hospital, Via M, Coppino 26, 12100 Cuneo, Italy; 3 Division of Cardiothoracic Surgery, College of Physicians and Surgeon of Columbia University - New York Presbyterian Hospital, New York, USA

## Abstract

**Background:**

Myasthenia gravis, an uncommon autoimmune syndrome, is commonly associated with thymus abnormalities. Thymomatous myasthenia gravis is considered to have worst prognosis and thymectomy can reverse symptoms if precociously performed.

**Case report:**

We describe a case of a patient who underwent mitral valve repair and was found to have an occasional thymomatous mass during the surgery. A total thymectomy was performed concomitantly to the mitral valve repair.

**Conclusion:**

The diagnosis of thymomatous myasthenia gravis was confirmed postoperatively. Following the surgery this patient was strictly monitored and at 1-year follow-up a complete stable remission had been successfully achieved.

## Background

Myasthenia gravis (MG), an uncommon autoimmune syndrome caused by the failure of neuromuscular transmission, results from binding of autoantibodies to those proteins that are involved in signaling at the neuromuscular junction [1].

The role of thymus in the pathogenesis of myasthenia gravis is not entirely clear, but most patients with myasthenia gravis are found to have some degree of thymus abnormality. The thymus is hypothesized to be the site of autoantibody formation and therefore thymectomy has been proposed as a first line therapy. This is especially true if a thymoma is present, as thymectomy has been reported to significantly improve the clinical condition [2].

## Case presentation

We report a case of a 37-year-old white female who presented with an echocardiographic diagnosis of severe mitral valve regurgitation and had a history of fatigue, weakness and dyspnea on exertion for last three months. Her symptoms were not further investigated considering the severe mitral valvular disease. She had no other co-morbidities and the preoperative EuroSCORE was 3. She was scheduled for an elective mitral valve repair surgery.

**Figure 1:**
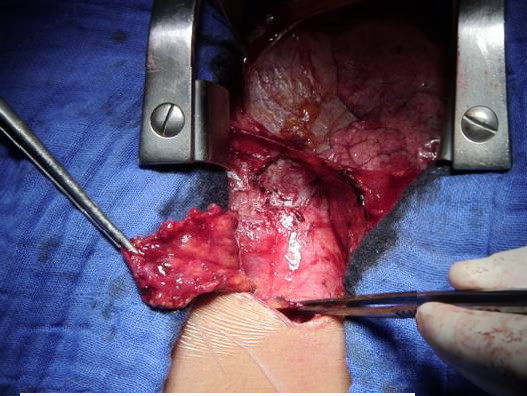
**The intraoperative finding of the small thymomatous mass which led to the MG diagnosis**.

The patient underwent routine median sternotomy. At direct inspection of the retro-sternal space, the superior third of the anterior mediastinum was filled with a 2 × 1.5 cm mass arising from left lobe of thymus (Figure 1). The mass was not invasive and easily resectable. No intraoperative frozen sections were examined. However, A total thymectomy was performed before opening the pericardium. Thereafter, the classic mitral valve repair was performed without any intraoperative or perioperative complication.

In the postoperative stay, high titers of anti-acetylcholine receptor antibodies and anti-striated muscle antibodies were found (9.1 nmol/L and titer >1:80, respectively) and myasthenia gravis was diagnosed. MG diagnosis was further confirmed by the positive tensilon test. The severity of MG was retrospectively evaluated in the postoperative period according to the clinical classification of the Medical Scientific Advisory Board of the Myasthenia Gravis Foundation of America (MGFA) [3]. The patient was classified to be in MGFA class IIa, as mild weakness involved not only ocular muscles but also axial muscles while oropharyngeal and respiratory muscles were not concerned. The histopathologic examination of the specimen revealed a completely excised thymoma. It was classified as type A accordingly to new World Health Organization classification of Rosai and Sabin (WHO type A or medullary). Using the staging process described by Masaoka, this specimen was staged to be as Masaoka Stage I since macroscopically it was a completely encapsulated thymoma with no microscopically determined capsular invasion [4].

The postoperative course was uneventful and the patient was discharged on postoperative day 6 to home-rehabilitation in tele-cardiology without any complications. The patient was strictly monitored by a multidisciplinary team composed of a cardiologist, a surgeon and a neurologist for regular follow-ups. No MG therapy was initiated considering the recent operation and the good clinical status. At six-month follow-up, no mitral regurgitation was detected by transthoracic echocardiography and the symptoms related to MG were completely remitted. The anti-acetylcholine receptor antibodies titer decreased to 4.2 nmol/L. At 1-year follow-up a complete stable remission (CSR) was assessed according to the MGFA Post-Intervention Status Classification and the acetylcholine receptor antibodies titer had further decreased to 1.4 nmol/L [3].

## Conclusion

Thymomatous MG (T-MG) is considered to have worst prognosis compared with non-thymomatous MG. The patho-physiological bases are not clear but clinical data suggest that patients with T-MG have high-grade symptoms with low rate of remission even after therapy [5].

In patients who undergo cardiac surgery, the evaluation of the thymus is often considered secondary and tumoral disease of the thymus is only considered when a mass is found intraoperatively or at a CT scan. Few reports have focused on the incidental finding of a thymic mass during cardiac surgery and the management is generally guided by the type and extension [6,7]. Total thymectomy is advised if an encapsulated thymic mass or a resectable invasive thymoma are found. However, histological examination on frozen section should be performed first if the malignant mass is unresectable or metastases are evident [8].

Moreover, the clinical evaluation usually represents the first diagnostic step for a patient with suspected MG. The evaluation of MG-related symptoms could be difficult as they may get masked by the cardiac disease. In this report, the preoperative clinical status was not correctly addressed as the cardiac symptoms were predominant. Hence, the diagnosis of thymomatous MG was guided by the intraoperative findings which led us to revaluate the preoperative clinical conditions.

Although uncommon, MG represents an invalidating disease which has to be diagnosed as soon as possible in order to initiate the appropriate therapy thereby increasing the remission rate [1,3,5]. The clinical evaluation should be more accurate in patients with cardiac disease as initial MG symptoms could be masked resulting in an underestimated or incorrect diagnosis. Moreover, the meticulous evaluation of the thymus gland itself during cardiac surgery can be an effective step towards finding even small macroscopic abnormalities of thymus that could be prophylactically excised. Therefore, a focus on thymus during cardiac surgery may not only lead to an occasional intraoperative diagnosis of thymus abnormality but also results in re-evaluation of the clinical status postoperatively to confirm the suspected concomitant T-MG.

## Consent

The written consent for publication was obtained. A copy of the written consent is available for review by the Editor-in-Chief of this journal.

## Competing interests

The authors declare that they have no competing interests.

## Authors' contributions

MA conceived the study idea, wrote the first draft and led the project from beginning to end. FB assisted the study in data collection, draft revision and coordinating with all co-authors. LD helped with literature review and manuscript writing. AC helped the study with discussions about the topic and assistance in manuscript writing. FHC edited the manuscript and helped with revisions and final submission. PB provided expert opinion throughout the study and also operated on this case. All authors read and approved the final manuscript.
